# Giant coronary aneurysm of Behcet’s disease with sudden syncope: a case report

**DOI:** 10.1186/s12872-023-03501-7

**Published:** 2023-09-15

**Authors:** Jingwei Feng, Qi Miao, Chaoji Zhang

**Affiliations:** 1 Research Center of Plastic Surgery Hospital, Chinese Academy of Medical Sciences and Peking Union Medical College, Beijing, 100144 PR China; 2 Department of Cardiac Surgery, Peking Union Medical College Hospital, Peking Union Medical College, Chinese Academy of Medical Sciences, Beijing, China

**Keywords:** Bedcet's disease, Coronary artery aneurysy, Syncope

## Abstract

Behcet’s disease(BD) is a chronic inflammatory vasculitis that rarely affects the arteries, making myocardial infarction unlikely. We report a 28-year-old patient who was admitted to our hospital with multiple sudden syncope. Cardiovascular risk factors such as hypertension (HT), diabetes and obesity were not found in her. Preoperatively, imaging examinations suggested thrombosis of the inferior and superior vena cava and right heart combined with coronary artery aneurysm. The patient was finally diagnosed with a huge coronary artery aneurysm proximal to the left anterior descending artery. Syncope is considered to be caused right ventricular outflow tract obstruction. The patient received a successful aneurysm resection and had an uneventful postoperative recovery.

## Introduction

BD is a chronic systemic inflammatory vasculitis with a wide range of clinical manifestations, including recurrent oral and genital ulcers; cutaneous lesions; and ophthalmic, neurologic, and gastrointestinal involvement [1]. Cardiac involvement is very rare but can occur with different presentations, including pericarditis, cardiomyopathy, endocarditis, endomyocardial fibrosis, intracavitary thrombosis, and coronary artery disease [2]. In fact, genetic predisposition and immune dysregulation leading to inflammation, endothelial damage, and impaired fibrinolysis contribute to its pathogenesis [3].

## Case Presentation

A 28-year-old woman was admitted to our hospital due to intermittent sudden syncope for half a year. In 2021-11, she experienced dizziness and lost consciousness and recovered spontaneously a few minutes later. Syncope occurred three times on 2022-2, 2022-4 and 2022-6-13, and the time of losing consciousness increased gradually. The last syncope lasted for half an hour, we gave the patient oxygen and fluid infusion treatment, and the patient gradually woke up. The patient had been diagnosed with BD for 6 years and had secondary right heart thrombosis and pulmonary artery thrombosis for 4 years; After receiving standard immunotherapy and anticoagulation therapy, symptoms and immune indexes of BD returned to normal, the discomfort symptoms of pulmonary embolism were improved, and the cardiac function was Grade I (NYHA).

## Image findings

**Fig. 1:**
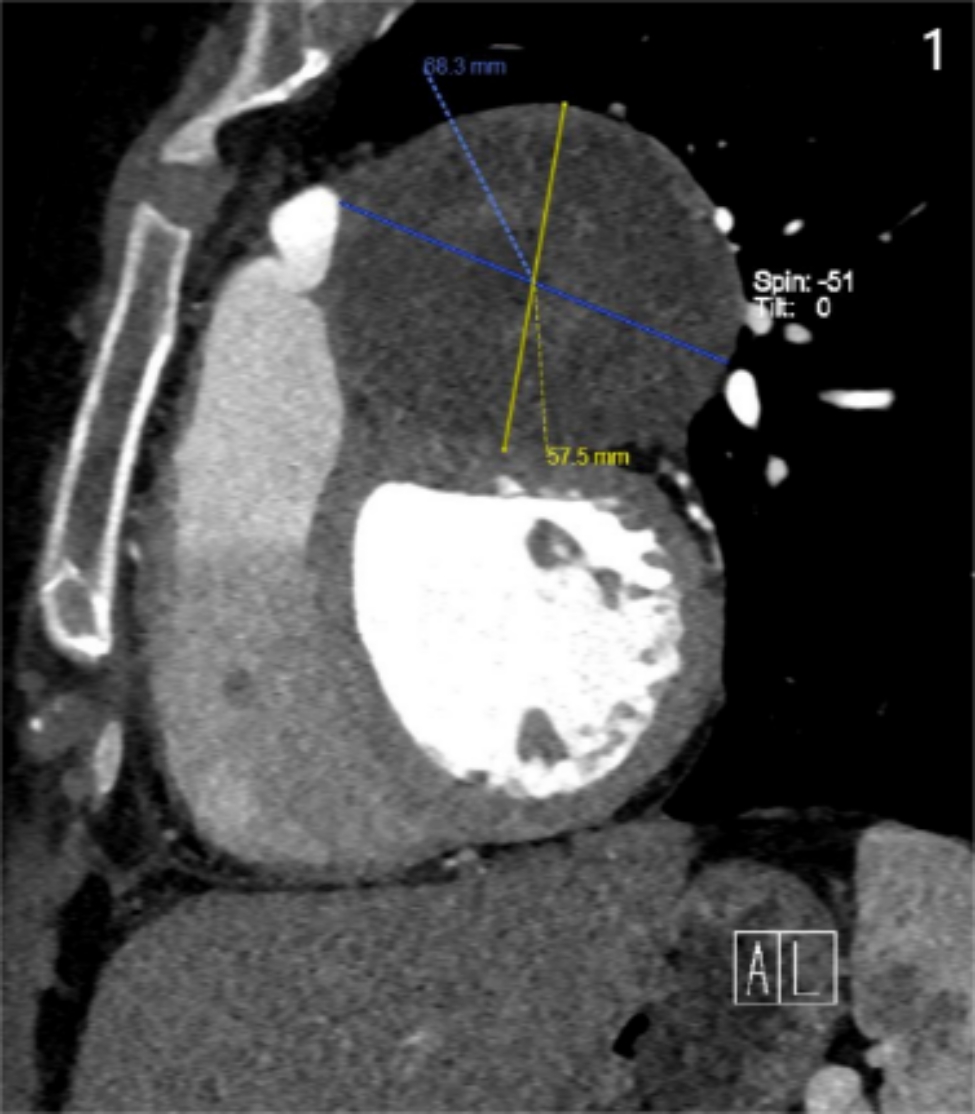
A computed tomographic scan of the coronary artery shows a round-shaped soft tissue density shadow, measuring approximately 6.8 × 5.7 cm

The patient’s Computed tomography angiogram of the pulmonary arteries (CTPA) and computed tomographic scan of the coronary artery revealed that in the pericardial cavity, a circular soft tissue shadow can be seen on the left side of the main pulmonary artery and left pulmonary artery, and on the left side of the aortic root, which is uneven in density and about 6.9 × 6. 5 cm in size. The mass compresses the left anterior descending branch. The patient had multiple previous pulmonary embolisms in the pulmonary segment, which was significantly improved compared to the imaging examination in 2018 (Fig. 1). Echocardiography suggests that there are many mural thrombosis in the right atrium, superior cavity, and inferior vena cava entrance, a round uneven shadow on the left side of the pericardium and beside the pulmonary trunk, with a size of about 7.3 × 6. 7 cm.

**Fig. 2:**
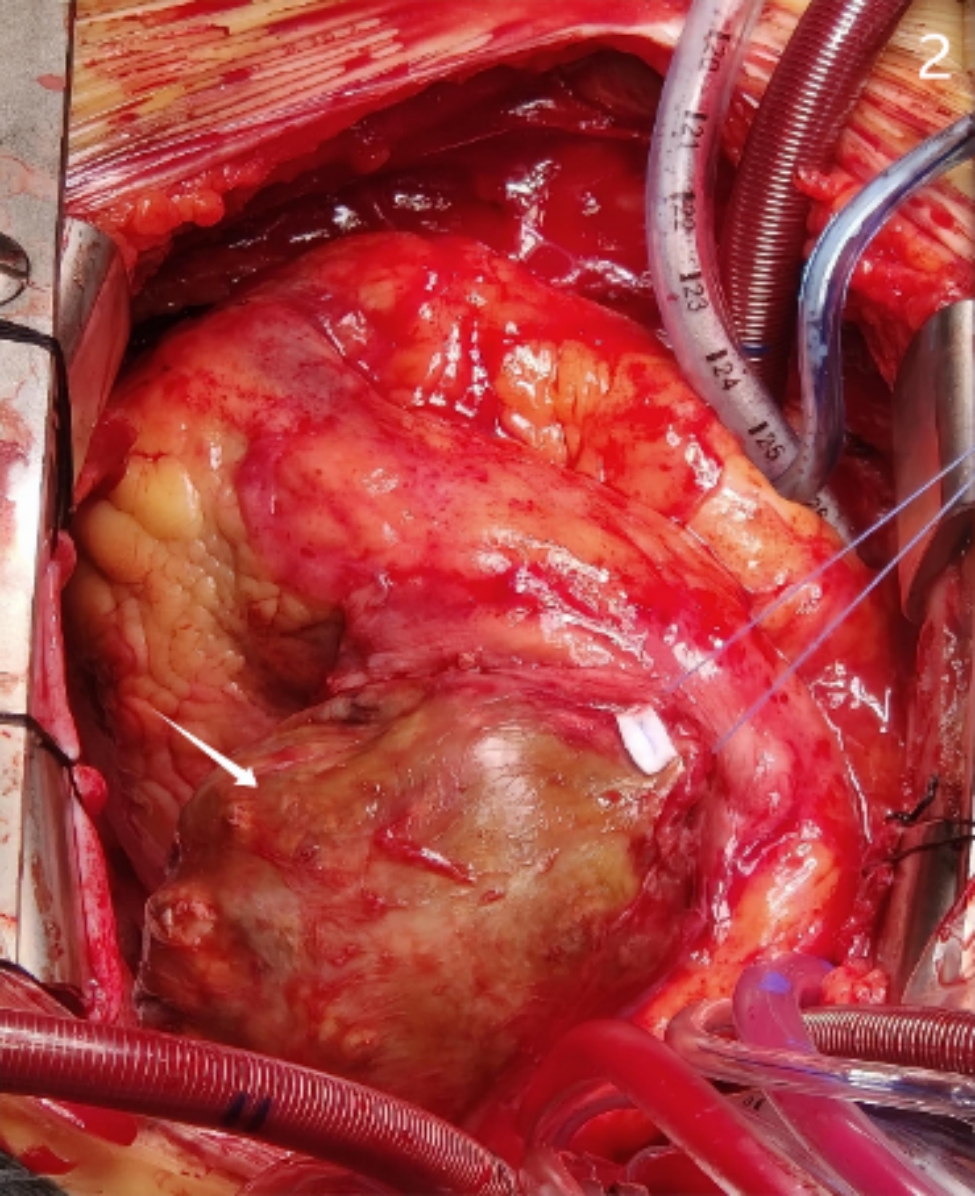
The mass is located on the left side of the left ventricle and the compression symptoms are obvious

Based on the above findings, the mass is considered a coronary artery aneurysm, and the huge mass can cause obstruction of the right ventricular outflow tract and has the risk of rupture. Given the risk of coronary aneurysm rupture, the patient did not undergo coronary angiography; On 4 July, 2022, a thoracotomy was performed under cardiopulmonary bypass. The mass was located in the pericardium, the left anterolateral side of the left ventricle, tightly adhered to the pericardium and was carefully dissociated. The size of the cystic tumor was 7 × 6 × 6 cm (Fig. 2). The anterior wall of the cystic cavity was incised and mural thrombus and blood components were visible there. Thrombus and blood were cleared, and cardioplegia was perfused. The posterior wall of the cyst was seen, with a fistula with a diameter of 3 mm overflowing perfusion fluid. A large number of thrombi were cleared and the fistula was intermittently sutured. and calcified thrombus tissue right atrium main pulmonary artery was Rewarming, opening Ao blocking forceps, automatic re-jumping. Get rid of cardiopulmonary bypass smoothly. Postoperation pathology showed that the tumor cyst wall was a component of blood vessel wall. Recovered well aft operation, discharged smoothly, and no syncope occurred.

## Discussion and conclusion

We report the patient with a diagnosis of BD who was treated with immunosuppressive therapy with complete remission of extrinsic systemic manifestations. In fact, there was no relationship between the severity of the cardiac lesions and that of the extracardiac manifestations of the disease [4]. Vascular involvement in BD is reported to be up to 40% in different series. The abdominal and thoracic aorta and pulmonary and femoral arteries are the most commonly involved arteries. However coronary arteries are rarely affected [5], occurring in 0.5% of cases [6]. Arterial aneurysm formation is characterized pathologically by neutrophilic vasculitis of the vasa vasorum, which in turn leads to destruction and necrosis of elastic fibers and smooth-muscle cells in the media [7]. It should be noted that most patients with coronary events were previously diagnosed with BD and were on regular treatment; less commonly, coronary complications may occur as the first manifestation of the disease [8]. Combined with the patient clinical manifestations, imaging studies, and intraoperative findings, the patient’s recurrent syncope was considered to be caused by compression of the right ventricular outflow tract due to the giant coronary aneurysm. Secondly, the coronary artery aneurysm in this patient is not visible in the coronary CTA. In fact, thrombosis may form in some coronary aneurysms and the tumor may not be visible on the coronary CTA, greatly increasing the difficulty of diagnosis [9]. Aneurysms in Behçet syndrome are often surrounded by a thick inflammatory fibrotic tissue (thought to prevent catastrophic free rupture) and hyperplastic lymph nodes that are different from those seen in atherosclerosis [10].

In fact, the patient we reported had thrombus formation in both the right atrium and the superior and inferior vena cava. Most reported cases show evidence of right cardiac thrombi, with the right ventricle being more affected than the atrium [11, 12]. Less frequently, thrombosis may be detected in the superior and inferior vena cava [13]. In BD, increased thromboembolism is triggered due to endothelial dysfunction, platelet activation, von Willebrand factor release, increased fibrin and thrombin release, and antithrombin deficiency [14]. Note that the decision and timing of intravascular or open surgery in Behcet’s aneurysm is controversial, Surgery can be complicated by occlusion or recurrence of the aneurysm, due to a pathergy-like vascular reaction to surgical trauma [15]. Although the preoperative diagnosis of the patient is somewhat biased. Fortunately, we used a median thoracotomy approach to remove the tumor, which allowed us to establish cardiopulmonary bypass safely and complete coronary artery aneurysm resection and thrombus clearance. Coronary artery aneurysms caused by BD have been reported in the past, most of which are asymptomatic coronary artery aneurysms, and it is very rare to have syncope as the first symptom. Because BD is easy to cause thrombosis, coronary artery aneurysms may not be typical in CTA.

The report highlights that coronary artery involvement in patients with BD may be characterized by syncope as the first manifestation. For patients who have already been diagnosed with BD, imaging tests should be performed regularly. Imaging is also necessary for patients whose extracardiac symptoms have been controlled.

## Data Availability

Not applicable.
